# Oriented, molecularly imprinted cavities with dual binding sites for highly sensitive and selective recognition of cortisol

**DOI:** 10.1098/rsos.170300

**Published:** 2017-08-16

**Authors:** Narito Suda, Hirobumi Sunayama, Yukiya Kitayama, Yuri Kamon, Toshifumi Takeuchi

**Affiliations:** Graduate School of Engineering, Kobe University, 1-1 Rokkodai-cho, Nada-ku, Kobe 657-8501, Japan

**Keywords:** molecular imprinting, stress marker, cortisol, molecular recognition, competitive binding assay, saliva

## Abstract

Novel, molecularly imprinted polymers (MIPs) were developed for the highly sensitive and selective recognition of the stress marker cortisol. Oriented, homogeneous cavities with two binding sites for cortisol were fabricated by surface-initiated atom transfer radical polymerization, using a cortisol motif template molecule (TM1) which consists of a polymerizable moiety attached at the 3-carbonyl group of cortisol via an oxime linkage and an adamantane carboxylate moiety coupled with the 21-hydroxyl group. TM1 was orientationally immobilized on a β*-*cyclodextrin (β-CD)-grafted gold-coated sensor chip by inclusion of the adamantane moiety of TM1, followed by copolymerization of a hydrophilic comonomer, 2-methacryloyloxyethyl phosphorylcholine, with or without a cross-linker, *N,N′*-methylenebisacrylamide. Subsequent cleavage of the oxime linkage leaves the imprinted cavities that contain dual binding sites—namely, the aminooxy group and β-CD—capable of oxime formation and hydrophobic interaction, respectively. As an application, MIP-based picomolar level detection of cortisol was demonstrated by a competitive binding assay using a fluorescent competitor. Cross-linking of the MIP imparts rigidity to the binding cavities, and improves the selectivity and sensitivity significantly, reducing the limit of detection to 4.8 pM. In addition, detection of cortisol in saliva samples was demonstrated as a feasibility study.

## 1. Introduction

Cortisol is a stress biomarker secreted from the adrenal gland in response to stress. Thus, the highly sensitive detection of cortisol has been used to diagnose mental disorders. Representative methods of detecting cortisol include enzyme-linked immunosorbent assay and mass spectrometry [1–4]. However, widespread use of these methods in self-medication and general healthcare facilities is limited by the high costs of unstable natural proteins, expensive instruments and complicated procedures.

Molecularly imprinted polymers (MIPs), known as polymer-based artificial receptors, have attracted significant attention because of their high stability, low cost and capabilities gained by further functionalization such as stimuli-responsiveness and signal transduction [5–13]. Typically, MIPs are synthesized by radical copolymerization of template molecules covalently or non-covalently conjugated with functional monomers, comonomers and cross-linking agents. The subsequent removal of the template molecules from the resulting polymers yields MIPs with specific binding cavities for the template molecules, which are complementary in size and shape.

Several cortisol-imprinted polymers have been previously reported [14,15]. They were typically prepared using a single functional monomer because plural binding sites in an imprinted cavity appeared to indicate more sensitive and selective binding activity towards target molecules such as nucleobases [16,17], endocrine desolators [18], herbicides [19], antibiotics [20,21], proteins [22–26], glycans [27–29], organic pollutants [30,31] and enzyme inhibitors [32,33]. We have reported cortisol-MIPs prepared with the ability to recognize cortisol using cortisol-21-monomethacrylate and itaconic acid as the template molecule and functional monomer, respectively. Moreover, a cortisol nanosensor platform based on the MIP particles and fluorescent-labelled cortisol was successfully demonstrated [34,35]. However, the limit of detection (*ca* 80 nM) was not sufficient for practical use.

Recently, an approach for the precise formation of oriented protein recognition cavities in MIP thin layers, with high affinity and selectivity towards the target protein, was developed using immobilized template protein molecules via well-defined protein–ligand interactions. The specific ligand served both as the protein-immobilizing agent during the surface-initiated controlled/living radical polymerization, and as the interaction site for the target protein after the construction of the imprinted binding cavity [36]. To date, this strategy can be applied only to proteins with binding sites for specific ligands, and further extension to other targets such as small molecules is still a challenging task. Thus, MIPs are highly specific and sensitive molecular recognition materials that can provide an alternative to natural antibodies for small molecules, which are not easily available.

**Figure 1.**
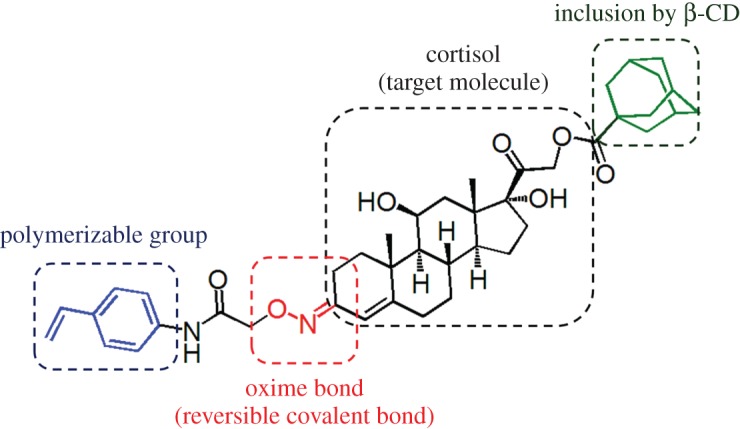
The TM1 template molecule, designed for cortisol imprinting.

In this study, a novel cortisol motif template molecule (TM1), cortisol-3-oxime-{*O*-[*N*-(4-vinyl-phenyl)]acetamide}-21-adamantane carboxylate (figure 1), was designed to construct MIP thin layers with orientated binding cavities that contained two interaction sites for cortisol. TM1 is composed of a cortisol molecule linked to 2-aminooxy-*N*-(4-vinylphenyl)acetamide at the 3-carbonyl group through reversible oxime bond formation [37–40] and to 1-adamantane carboxylic acid at the 21-hydroxyl group through ester formation. The adamantane moiety can interact with β-cyclodextrin (β-CD) by hydrophobic inclusion [41–43]. The reversible oxime linkage enables the removal of TM1 from the cortisol-imprinted cavity, and the resulting aminooxy group can serve as the interaction site for the 3-carbonyl group of cortisol. The adamantyl group induces the orientational immobilization of TM1 on a β-CD-grafted gold-coated glass substrate by hydrophobic interaction. The polymerization is conducted using 2-methacryloyloxyethyl phosphorylcholine (MPC) as a biocompatible comonomer, and subsequent removal of TM1 by hydrolysis of the oxime bond followed by washing with ethanol leaving the aminooxy group and the β-CD moiety within the imprinted cavity. Surface-initiated atom transfer radical polymerization using activators generated by electron transfer (SI-AGET ATRP) [44–50] is employed to form a homogeneous polymer matrix around the imprinted cavities, resulting in MIP thin layers with the desired thickness and low non-specific binding properties [51]. Herein, a highly sensitive cortisol detection technique was developed based on a fluorescence competitive binding assay using a fluorescent-labelled competitor in standard aqueous solutions and saliva samples. This method demonstrates that oriented, molecularly imprinted cavities with dual binding sites have significant potential as a powerful, cost-effective and rational tool to provide a synthetic alternative to natural antibodies to low molecular weight compounds.

## 2. Results and discussion

**Scheme 1.**
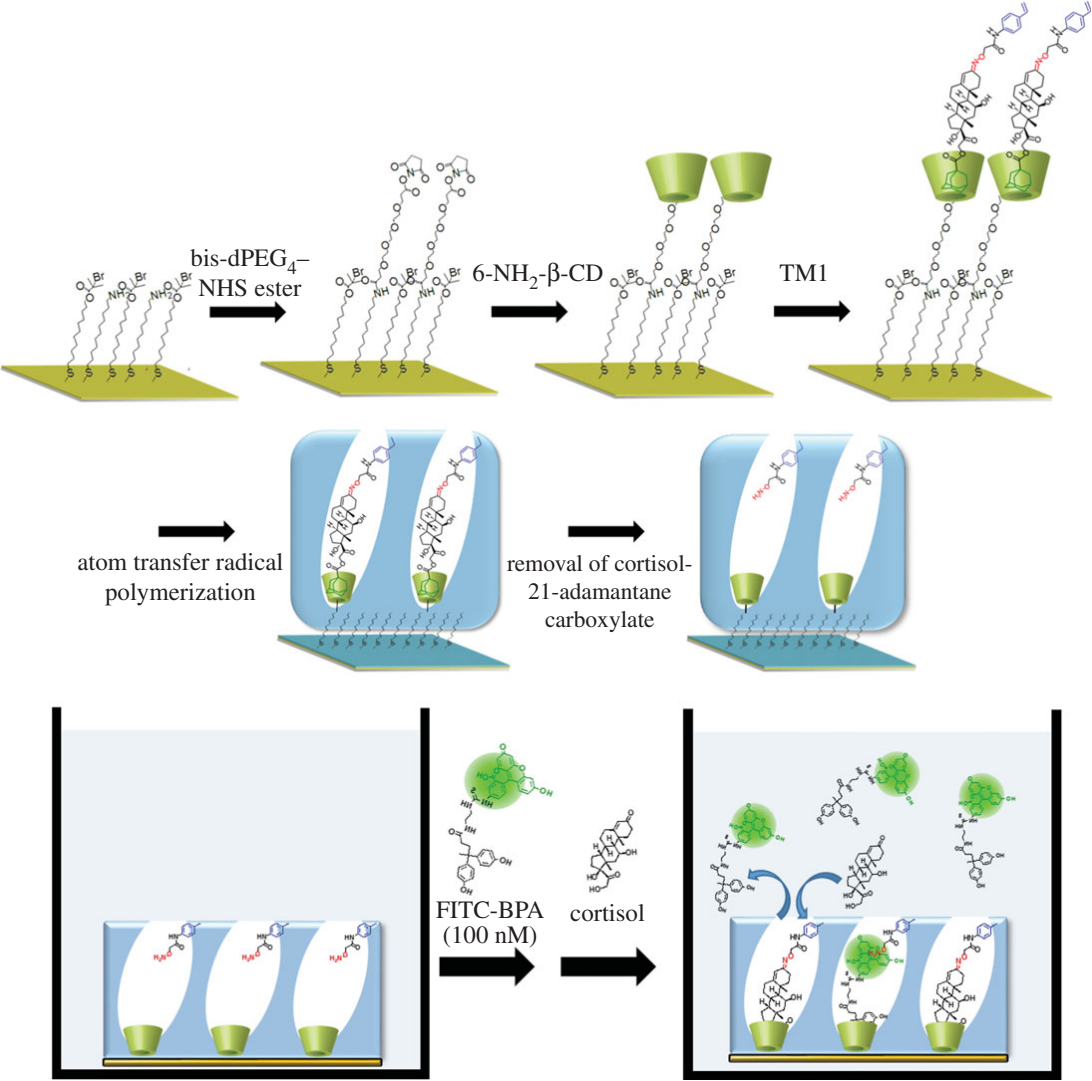
Preparation of the cortisol-MIP thin layer containing oriented cavities with dual binding sites within the imprinted cavity (*a*) and schematic of the competitive binding assay using FITC-BPA as the fluorescent competitor (*b*).

TM1 consists of cortisol-21-adamantane carboxylic acid ester, which is capable of forming inclusion complexes with β-CD, linked with a polymerizable aminooxy moiety at the 3-carbonyl group of cortisol through a reversible oxime bond (figure 1; electronic supplementary material, scheme S1). A mixed, self-assembled monolayer (SAM) is formed on the surface of a gold-coated glass substrate to introduce amino and bromo groups (initiators of SI-AGET ATRP) on the surface (scheme 1*a*). In order to orientationally immobilize TM1 on the substrate, 6-NH_2_-β-CD; (electronic supplementary material, scheme S2) is coupled with the amino groups on the substrate via bis-dPEG_4_-NHS ester. The interaction between β-CD and the adamantyl group of TM1 was confirmed by ^1^H-NMR (electronic supplementary material, figure S1), while no interaction was observed between β-CD and the styryl group of TM1. The subsequent immobilization of TM1 is carried out by inclusion of the TM1 adamantane moiety into the immobilized β-CD in aqueous solution. The oriented immobilization of TM1 can be achieved because of preferable inclusion of the 21-adamantane moiety compared with the *N*-(4-vinylphenyl)acetamide moiety. According to previous reports, such oriented immobilization enables the construction of homogeneous binding cavities, unlike conventional MIP preparation techniques [52–55]. Next, SI-AGET ATRP of MPC was conducted on the TM1-conjugated gold-coated glass substrate, with or without *N,N′*-methylenebisacrylamide (MBAAm) addition and using CuBr_2_/*N,N,N′,N′′,N′′*-pentamethyldiethylenetriamine (PMDETA) as the catalyst. The phospholipid-mimetic, biocompatible MPC monomer [56] was chosen as the comonomer to make the polymer matrix hydrophilic and to reduce non-specific binding of off-target proteins, which may interfere with cortisol recognition in real samples. After polymerization, TM1 was removed by cleaving the oxime linkage by acid hydrolysis [57]. The SAM and the MPC polymer matrix are confirmed to be stable under the acidic conditions employed (electronic supplementary material, table S1). In order to confirm formation of the MIP thin layer on the substrate via SI-AGET ATRP, elemental analysis was conducted using X-ray photoelectron spectroscopy. After polymerization, a P 2*p* peak with a maximum at around 134 eV is observed, and is attributed to the phosphorylcholine group (electronic supplementary material, figure S2), which indicates that the poly(MPC) layer was successfully formed on the gold-coated glass substrate. In addition, X-ray reflectometry measurements were performed to evaluate the thickness of the MIP thin layer (electronic supplementary material, figure S3 and table S2). After 30 min of polymerization, the thickness is estimated to be approximately 3 nm, and the polymerization proceeds linearly with time, indicating that the MIP thin layer formation takes place in a living manner (electronic supplementary material, figure S4).

The binding activity of the MIP thin layers towards cortisol is evaluated by a competitive binding assay (scheme 1*b*). Because bisphenol A (BPA) is known as a possible endocrine disruptor and can bind to glucocorticoid receptors [58,59], fluorescein-conjugated BPA (FITC-BPA) is prepared as a novel fluorescent competitor for cortisol binding (electronic supplementary material, scheme S3). Since a possible complex of FITC-BPA and β-CD (approx. 20 Å) is estimated to be the same size as TM1 and β-CD (electronic supplementary material, figure S5) via a molecular mechanics-based docking simulation under aqueous conditions (dielectric constant = 80), FITC-BPA is expected to be an accessible competitor for the imprinted cavity. In the competitive binding assay, FITC-BPA first interacts with the MIP, then adds cortisol, resulting in desorption of FITC-BPA from the binding cavities depending on the concentration of cortisol added. In order to confirm whether FITC-BPA functions as a competitor, i.e. has a moderate affinity for cortisol binding sites, FITC-BPA (final concentration 0–400 nM) was incubated in the presence of the MIP thin layer in a 10 mM phosphate buffer (pH 7.4). The incubation time was set to 20 min, which allows a constant fluorescence intensity value (electronic supplementary material, figure S6). The fluorescence intensity derived from unbound FITC-BPA in the supernatant was measured. A fluorescence intensity change (Δ*I* *=* *I* − *I*_0_) was used as an index for identification of binding events (*I*_0_ and *I* are defined as the fluorescence intensities before and after the incubation of FITC-BPA with the MIP thin layer, respectively). The Δ*I* values decrease with the addition of FITC-BPA, indicating that FITC-BPA is able to bind to the cortisol binding cavities in the MIP thin layer, and that the binding cavities are almost saturated with 100 nM FITC-BPA (electronic supplementary material, figure S7). It should be noted that no fluorescence change is detected upon interaction between free cortisol and free FITC-BPA. When various concentrations of cortisol are added to the buffer solution containing 100 nM FITC-BPA, no fluorescence change is observed (electronic supplementary material, figure S8), confirming that cortisol does not interact with FITC-BPA during the competitive binding assay.

**Figure 2.**
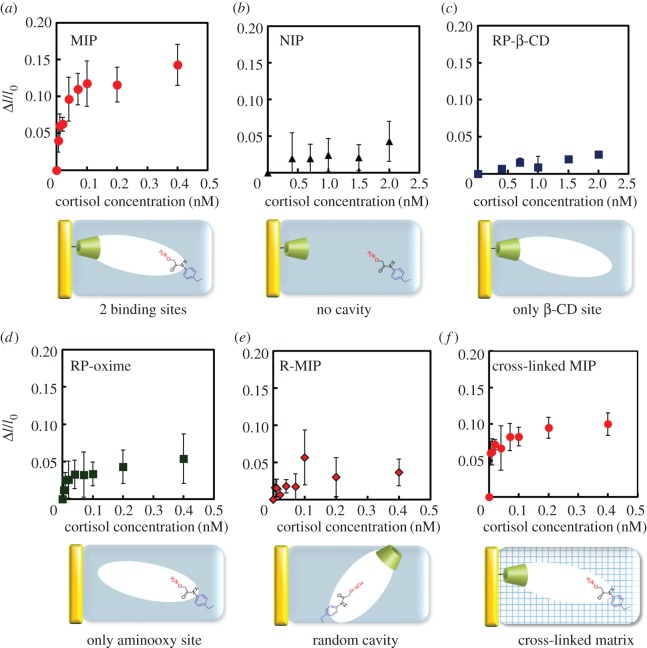
Competitive cortisol binding and FITC-BPA (100 nM) to MIP (*a*), NIP (*b*), RP-β-CD (*c*), RP-oxime (*d*), R-MIP (*e*) and cross-linked MIP (*f*) in 10 mM phosphate buffer (pH 7.4).

In order to examine the binding behaviour of cortisol towards the MIP thin layer, fluorescence measurements of the FITC-BPA released by competitive cortisol binding were performed (figure 2*a*). The MIP was immersed in the buffer solution containing 100 nM FITC-BPA, and various amounts of cortisol (final concentration 0–0.4 nM) were added, followed by incubation for 20 min. The relative fluorescence intensity change (Δ*I*/*I*_0_) caused by the FITC-BPA released from the MIP thin layer because of the competitive binding was measured. The incubation time was set at 20 min, because the Δ*I*/*I*_0_ value remained constant 20 min after the addition of cortisol (electronic supplementary material, figure S9). The Δ*I*/*I*_0_ values in the supernatant increase with the cortisol concentration, and the poly(MPC) itself (only the polymer matrix) does not respond to the competitive binding assay under the same conditions described above (electronic supplementary material, figure S10).

The apparent binding constant (*K*_a_) of the non-cross-linked MIP thin layer is estimated to be 1.28 × 10^11^ M^−1^ by curve fitting using DeltaGraph v. 5.4.5 (electronic supplementary material, figure S11*a*). The limit of detection is 7.1 pM, as calculated using 3 s.d.*/m* (*m*: slope of the linear part of the binding isotherm, s.d.: standard deviation) [60], which confirms that the imprinting process enables installation of the aminooxy group and the β-CD moiety in a position more favourable for the specific cortisol binding.

A non-imprinted polymer (NIP) with randomly located aminooxy groups and β-CD-immobilized on the substrate is prepared without the use of TM1 (no imprinted cavity for cortisol; electronic supplementary material, scheme S4). Binding NIP experiments are conducted and compared with the results obtained for MIP (figure 2*a*): the Δ*I/I*_0_ values for NIP are very small (figure 2*b*) and the apparent *K*_a_ (3.93 × 10^10^ M^−1^) is 3.3 times smaller than that of MIP (electronic supplementary material, figure S11*b*), revealing that the TM1 imprinting process induces a high affinity and, therefore, confirming the efficiency of the imprinting effect.

In order to evaluate the effectiveness of the dual binding sites in the imprinted cavity, two reference MIP thin layers with only one binding site to either the immobilized β-CD (RP-β-CD; electronic supplementary material, scheme S5) or the aminooxy group (RP-oxime; electronic supplementary material, scheme S6) in the imprinted cavity were prepared. The Δ*I/I*_0_ values for RP-β-CD (figure 2*c*) and RP-oxime (figure 2*d*) are also clearly smaller than those for MIP, and the apparent *K*_a_ values are 180 times (6.99 × 10^8^ M^−1^; electronic supplementary material, figure S11*c*) and 3 times (4.00 × 10^10^ M^−1^; electronic supplementary material, figure S11*d*) smaller than that of MIP, respectively. Based on these results, the dual binding sites are effective in developing a strong affinity towards cortisol. The effect of orientation of the imprinted cavities in MIP was proved by comparison with R-MIP containing randomly located cortisol-imprinted cavities with dual binding sites (electronic supplementary material, scheme S7), which was prepared using *N*-methacryloyl 6-amido-β-CD (6-MAm-β-CD; electronic supplementary material, scheme S2) and TM1. The Δ*I/I*_0_ values for R-MIP (figure 2*e*) were again smaller than those for MIP, and the apparent *K*_a_ was found to be three times smaller than that of MIP (4.01 × 10^10^ M^−1^; electronic supplementary material, figure S11*e*), confirming that the orientation of the imprinted cavities can enhance the affinity of MIP towards cortisol.

Because cross-linking of the polymer matrices was reported to affect the affinity and selectivity of the resultant MIPs for proteins [61], a cross-linked MIP is prepared using MBAAm as a hydrophilic cross-linker with 20 mol% of MPC (figure 2*f*). The apparent *K*_a_ value of the cross-linked MIP is estimated to be 2.30 × 10^11^ M^−1^ (electronic supplementary material, figure S11*f*), and the limit of detection is 4.8 pM. Thus, cross-linking during the imprinting process can enhance the affinity, which becomes comparable to that of antibodies [61,62]. The level of error appeared to decrease when the MIP was cross-linked, suggesting that reproducibility is influenced by the stability of the MIP surface, and can be improved by optimizing the cross-linker ratio and/or screening the cross-linking agents.

**Figure 3.**
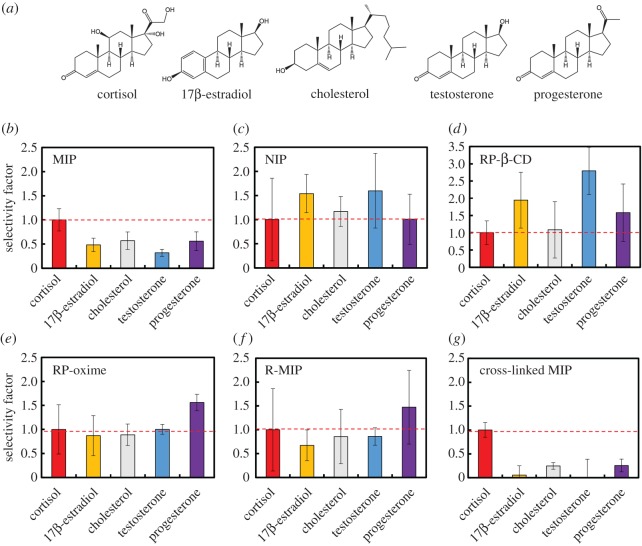
Chemical structures of cortisol and its tested structural analogues (*a*), and selectivities of MIP (*b*), NIP (*c*), RP-β-CD (*d*), RP-oxime (*e*), R-MIP (*f*) and cross-linked MIP (*g*) towards cortisol, 17β-estradiol, cholesterol, testosterone and progesterone. The selectivity factor was obtained from the ratio of (Δ*I*/*I*_0_)_reference compound_ to (Δ*I*/*I*_0_)_cortisol_.

In order to evaluate the effect of cross-linking on selectivity, the selectivities of MIP, NIP, RP-β-CD, RP-oxime, R-MIP and cross-linked MIP towards structural analogues of cortisol (figure 3*a*), such as 17β-estradiol (phenolic and aliphatic hydroxyl groups at C3 and C17, respectively), cholesterol (hydroxyl group at C3), testosterone (keto and hydroxyl groups at C3 and C20, respectively) and progesterone (two ketone moieties), were examined. The selectivity factor, defined as the ratio between (Δ*I*/*I*_0_)_reference compound_ and (Δ*I*/*I*_0_)_cortisol_, was used as an index of the selective binding capability. A value of less than 1 indicates high selectivity towards cortisol.

For MIP, the selectivity factors for all reference compounds are less than 1; the values of 0.48, 0.57, 0.32 and 0.56 are for 17β-estradiol, cholesterol, testosterone and progesterone, respectively (figure 3*b*), whereas NIP shows non-specific binding behaviour, with values of 1 or higher: 1.54, 1.17, 1.60 and 1.01 for 17β-estradiol, cholesterol, testosterone and progesterone, respectively (figure 3*c*). The lower selectivity of NIP towards cortisol demonstrates the influence of the imprinting effect. RP-β-CD shows a similar trend to that of NIP, with higher binding activity for 17β-estradiol (1.94), testosterone (2.79) and progesterone (1.58) than cortisol (figure 3*d*). This may be due to hydrophobic interactions with β-CD and to the more flexible side chains of cortisol and cholesterol, which may interfere with inclusion. RP-oxime and R-MIP show almost no selectivity (figure 3*e,f*), suggesting that the aminooxy group cannot form an oxime bond without the cooperative binding of β-CD, as also demonstrated by the fact that 17β-estradiol and cholesterol, which have no keto group, were bound in a similar manner as the other compounds. Thus, precise molecular recognition of cortisol is attributed to a dual point binding based on oxime formation and inclusion by β-CD.

Interestingly, as described above, the cross-linked MIP exhibits not only enhanced affinity but also remarkably higher selectivity towards cortisol than the other polymer matrices (figure 3*g*), with almost no cross-reactivity for testosterone and a 17β-estradiol selectivity factor that is eight times smaller. These results reveal that the cross-linked MIP can distinguish the slightly different side chains of steroidal compounds. Moreover, cross-linking dramatically improves the sensitivity and selectivity of the MIP, increasing the rigidity of the binding cavity. In addition, the orientational assembly of the recognition cavities greatly affects their selectivity, as demonstrated by the significant non-specific binding of progesterone in the R-MIP.

In order to assess whether the proposed protocol can be applied to cortisol detection in real samples, a competitive fluorescent binding assay was conducted using the cross-linked MIP in a 10 vol% saliva solution. The Δ*I*/*I*_0_ value clearly increases (electronic supplementary material, figure S12) with the cortisol concentration (*K_a_* = 4.47 × 10^10^ M^−1^), and saturation is observed at approximately 0.6 nM, where the detectability is comparable to that of commercially available ELISA [63] and radioimmuneassay [64]. Although the Δ*I*/*I*_0_ value is smaller than that obtained in the buffer solution (electronic supplementary material, figure S12), the apparent sensitivity is high enough to detect the cortisol secreted in human saliva (greater than 2 nM) [63]. The results clearly indicate that the MIP thin layer with oriented homogeneous cavities that contain dual binding sites developed herein exhibits effective cortisol recognition, and the highly sensitive detection of cortisol is confirmed by a competitive fluorescence assay using a real saliva sample.

## 3. Experimental set-up

### 3.1. Preparation of the mixed self-assembled monolayer on the gold-coated glass substrate

A gold-coated glass substrate was washed with water and EtOH, dried with N_2_, and then cleaned using a UV-O_3_ cleaner (Bioforce Nanosciences) for 20 min. The substrate was then immersed in an EtOH solution (5 ml) containing bis[2-(2-bromoisobutyryloxy)undecyl]disulfide (1.5 µl, 2.5 µmol) and 11-amino-1-undecanethiol hydrochloride (600 µg, 2.5 µmol) at 25°C for 24 h.

### 3.2. Immobilization of 6-NH_2_-β-CD on the mixed self-assembled monolayer-modified substrate

The mixed SAM-modified substrate was immersed in a dimethylformamide (DMF) solution (5 ml) containing bis-dPEG_4_-NHS ester (12.2 mg, 25 µmol) at 25°C for 1 h. The resulting dPEG_4_-NHS-grafted substrate was then immersed in a DMF solution (5 ml) containing 6-NH_2_-β-CD (17.0 mg, 15 µmol) at 25°C for 3 h.

### 3.3. Preparation of molecularly imprinted polymers via SI-AGET ATRP

TM1 (3.50 mg, 5 µmol) was dissolved in dimethylsulfoxide (200 µl), and pure water (5 ml) was added. The β-CD-immobilized substrate was immersed in the above solution (5 ml), and incubated for 24 h at 25°C, and then washed with pure water and dried with N_2_. A pre-polymerization solution (5 ml) containing CuBr_2_ (1.34 mg, 6 µmol), PMDETA (1.25 µl, 6 µmol) and MPC (88.6 mg, 300 µmol) was added into a Schlenk flask, and a TM1-conjugated β-CD-immobilized substrate was inserted. To prepare the cross-linked MIP, MBAAm (9.25 mg, 60 µmol) was added to the above pre-polymerization solution. Degassing and nitrogen substitution were repeated, and then ascorbic acid (0.52 mg, 3 µmol) dissolved in degassed water (1.0 ml) was injected. SI-AGET ATRP was conducted for 30 min at 25°C. After polymerization, the substrate was washed with pure water and immersed in a 1 M EDTA-4Na aqueous solution for 12 h at 25°C to remove Cu(II) ions. The substrate was then immersed into a 100 mM HCl aqueous solution for 12 h at 40°C to remove the cortisol-21-adamantane carboxylate moiety by hydrolysing the oxime bond. The substrate was washed with pure water and EtOH, and dried with N_2_.

### 3.4. Fluorescence-based competitive binding assay for cortisol using molecularly imprinted polymers and reference polymers

FITC-BPA (100 nM) dissolved in a 10 mM phosphate buffer (pH 7.4) was transferred into vials (5 ml), into which the polymer-coated substrates (MIPs and NIP) were inserted. The vials were incubated for 1 h at 25°C. Cortisol dissolved in a 10 mM phosphate buffer (pH 7.4) (final concentrations: 0 nM–0.4 nM for MIP, cross-linked MIP, RP-oxime, and R-MIP; 0–2 nM for RP-β-CD and NIP) was added. After 20 min at 25°C, the fluorescence intensities of the supernatants derived from free FITC-BPA that had been replaced by cortisol were measured (excitation wavelength: 495 nm, emission wavelength: 515 nm). For the selectivity experiments, structural analogues 17β-estradiol, cholesterol, testosterone and progesterone (0.2 nM for MIP, cross-linked MIP, RP-oxime and R-MIP; 1.5 nM for RP-β-CD and NIP) were employed as reference compounds.

### 3.5. Recovery tests for cortisol from diluted saliva samples

Saliva samples were collected by Saliva Collection Aid (Salimetrics, Inc., USA) and diluted to 11% (v/v) with a 10 mM phosphate buffer (pH 7.4). FITC-BPA was then added to the saliva samples (100 nM). The cross-linked MIP-coated substrate was immersed in the sample solutions and incubated for 1 h. Cortisol dissolved in the phosphate buffer (50 µl) was added (final concentrations: 0–1.1 nM, total volume: 1 ml), and incubated for 20 min at 25°C. Finally, fluorescence was measured in the supernatants (excitation wavelength: 495 nm, emission wavelength: 515 nm).

## 4. Conclusion

In this study, a novel template molecule (TM1) was designed for the preparation of MIP thin layers that are highly selective towards cortisol and possess oriented homogeneous cavities with dual binding sites. TM1 was copolymerized with MPC in the absence and presence of the cross-linker MBAAm via SI-AGET ATRP on a β-CD-immobilized gold-coated glass substrate. Highly sensitive detection of cortisol using the MIP thin layers was successfully demonstrated by a simple fluorescence-based competitive binding assay: the apparent limits of detection were 7.1 pM (without cross-linking: *K*_a_ = 1.28 × 10^11^ M^−1^) and 4.8 pM (with cross-linking: *K*_a_ = 2.30 × 10^11^ M^−1^) in aqueous solution, suggesting that the rigidity of the imprinted cavities induced a more precise recognition towards cortisol. The binding experiments using reference matrices, namely, NIP, RP-β-CD, RP-oxime and R-MIP, showed that the dual interaction in the imprinted cavity, that is, the hydrophobic interaction with β-CD and oxime formation with the aminooxy residues in the MIP thin layer, provided significantly high affinity and selectivity for cortisol. Sensitive cortisol detection was also demonstrated in real saliva samples. We believe that the proposed MIP preparation strategy can facilitate the application of MIPs not only for the diagnosis of mental disorders but also for other bio/chemical sensing tasks in the field of life sciences. This approach can pave the way for the development of artificial receptors capable of molecular recognition comparable with that of natural antibodies.

## Supplementary Material

Supplementary material from Oriented, Molecularly Imprinted Cavities with Dual Binding Sites for Highly Sensitive and Selective Recognition of Cortisol

## Data accessibility

All the relevant data is presented in this paper and in the electronic supplementary information.
