# Supplementary material for: Oriented, molecularly imprinted cavities with dual binding sites for highly sensitive and selective recognition of cortisol
Source: R Soc Open Sci. 2017 Aug 16;4(8):170300. doi: 10.1098/rsos.170300 (PMC5579094; doi:10.1098/rsos.170300)
Supplement: Supplementary material from Oriented, Molecularly Imprinted Cavities with Dual Binding Sites for Highly Sensitive and Selective Recognition of Cortisol [file rsos170300supp1.pdf]

Supporting Information for

Oriented, Molecularly Imprinted Cavities with Dual Binding Sites for Highly Sensitive and Selective Recognition of Cortisol

Narito Suda, Hirobumi Sunayama, Yukiya Kitayama, Yuri Kamon, Toshifumi Takeuchi*
Graduated School of Engineering, Kobe University, 1-1 Rokkodai-cho, Nada-ku, Kobe 657-8501, Japan
Tel/Fax: +81-78-803-6158, E-mail: takeuchi@gold.kobe-u.ac.jp

Table of Contents

1. Materials 2
2. Characterization 2
3. Synthesis of the designed template molecule TM1 3
4. Synthesis of 6-amino-*β*-cyclodextrin (6-NH_2-*β*-CD) and *N*-methacryloyl 6-amido-*β*-cyclodextrin (6-MAm-*β*-CD) 5
5. Synthesis of fluorescein-labeled bisphenol A (FITC-BPA) 7
6. Stability of the SAM and the *poly*(MPC) on the gold-coated glass substrates 10
7. Preparation of a NIP thin layer for examination of the imprinting effect 11
8. Preparation of an RP-*β*-CD thin layer for examination of the effect of two point cortisol binding 12
9. Preparation of an RP-oxime thin layer to examine the effect of two point cortisol binding 13
10. Preparation of an R-MIP thin layer to examine the effect of orientational immobilization of TM1 14
11. Preparation of a *poly*(MPC) thin layer 14
12. Interaction between the adamantyl group of TM1 and *β*-CD 15
13. XPS measurements 16
14. XRR measurements and the estimation of polymer thickness 16
15. Estimation of possible *β*-CD complex sizes with FITC-BPA and TM1 19
16. Time course of FITC-BPA adsorption to MIP on the substrate 20
17. Adsorption of FITC-BPA by MIP 21
18. Interaction between FITC-BPA and cortisol 22
19. Time course of the replacement of FITC-BPA with cortisol 23

20. Fluorescence-based competitive binding assay for cortisol using MIPs, NIP, and a *poly*(MPC) thin layer 24

1. Materials

Sodium chloride (NaCl), sodium citrate, sodium hydrogen carbonate ($NaHCO_3$), potassium carbonate (K_2CO_3), sodium sulfate (Na_2SO_4), ethyl acetate (EtOAc), dichloromethane (CH_2Cl_2), ethylenediamine, fluorescein isothiocyanate, *N,N*-dimethyl-4-aminopyridine (DMAP), *p*-toluenesulfonyl chloride, sodium azide (NaN_3), copper (II) bromide ($CuBr_2$), L-ascorbic acid, and tetrasodium ethylenediaminetetraacetate (EDTA 4Na) were purchased from Nacalai Tesque (Kyoto, Japan). Methanol (MeOH), ethanol (EtOH), hexane (Hex), dimethyl sulfoxide (DMSO), acetonitrile (MeCN), *N,N*-dimethylformamide (DMF), sodium hydroxide (NaOH), acetic anhydride, disodium hydrogenphosphate, triphenylphosphine (Ph_3P), *N,N'*-methylenebisacrylamide (MBAAm), cholesterol, and testosterone were purchased from Wako Pure Chemical Industries (Osaka, Japan). Diphenolic acid, 1-ethyl-3-(3-dimethylaminopropyl) carbodiimide hydrochloride (EDC), 4-aminostyrene, 4-(4,6-dimethoxy-1,3,5-triazin-2-yl)-4-methylmorpholinium chloride (DMT-MM), pyrrolidine, *β*-cyclodextrin (*β*-CD), carboxymethoxylamine hemihydrochloride, and 17*β*-estradiol were purchased from Tokyo Chemical Industries, Co., Ltd. (Tokyo, Japan). Cortisol, bis [2-(2-bromoisobutyryloxy)-undecyl] disulfide, *N,N,N',N",N"*-pentamethyldiethylenetriamine (PMDETA), and progesterone were purchased from Sigma-Aldrich Japan (Tokyo, Japan). 1-Adamantanecarboxylic acid and sodium dihydrogenphosphate were purchased from KATAYAMA Chemical Industries CO., LTD. (Osaka, Japan). 4 M HCl in dioxane was purchased from Watanabe Chemical Industries (Hiroshima, Japan). *Di-tert*-butyl dicarbonate ($(Boc)_2$ O) was purchased from Peptide Institute Inc. (Osaka, Japan). 11-Amino-1-undecanethiol hydrochloride was purchased from DOJINDO LABORATORIES (Kumamoto, Japan). Bis (2,5-dioxopyrrolidin- 1-yl)-4,7,10,13-tetraoxahexadecane-1,16-dioate (Bis-dPEG$_4$-NHS ester) was purchased from Quanta BioDesign, Ltd. (Montgomery, USA). Deionized water used was obtained from a Millipore Milli-Q purification system. 2-Methacryloyloxyethyl phosphorylcholine (MPC) was purchased from NOF Corporation (Tokyo, Japan). Gold-coated glass substrates were purchased from JASCO Corporation (Tokyo, Japan). Saliva Collection Aid (SCA) and Salivary EIA Kit were purchased from Salimetrics, Inc. (Carlsbad, USA).

2. Characterization

^{1}H-NMR spectra were measured using a 300 MHz FT-NMR system (JNM-LA300 FT NMR system, JEOL Ltd., Tokyo, Japan). MALDI-TOF-MS measurements were conducted using

a Voyager-1000DE system (Applied Biosystems, USA). XPS measurements were conducted using a PHI X-tool system (ULVAC PHI, Inc., Kanagawa, Japan) at a takeoff angle of 45° and X-ray condition of 20 kV and 98 W with Al Kα. Fluorescence measurements were conducted using an F-2500 fluorescence spectrophotometer (Hitachi High-Technologies, Tokyo, Japan). Polymeric layer thicknesses were measured by XRR (SmartLab 3 kW, Tokyo, Japan).

3. Synthesis of the designed template molecule TM1

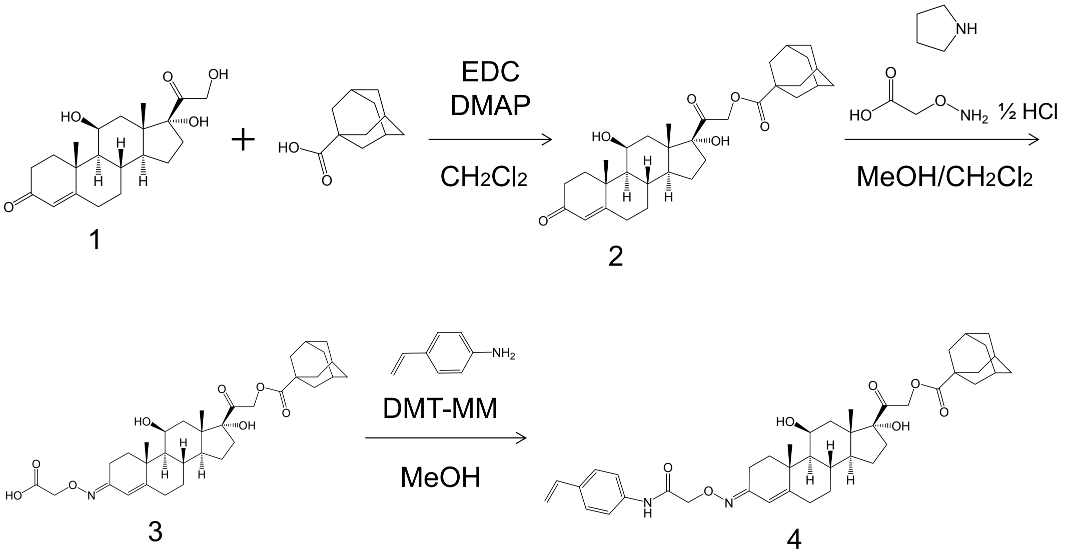

Scheme S1. Synthesis of TM1 for the imprinting of cortisol.

3-1. Cortisol-21-adamantane carboxylate (2)

1-Adamantane carboxylic acid (135 mg, 0.75 mmol), EDC (143 mg, 0.75 mmol), and DMAP (92 mg, 0.75 mmol) were dissolved in CH_2Cl_2 (5 mL) and stirred for 30 min under cooling with ice. Compound 1 (cortisol, 181 mg, 0.5 mmol) was then added, and the mixture was stirred overnight. The reaction solution was washed with a saturated citric acid aqueous solution, saturated $NaHCO_3$ aqueous solution, and brine. The crude product was purified using preparative chromatography (AI-580S, Yamazen Corporation, Osaka, Japan) (silica gel, Hex : EtOAc = 1 : 1). The solvent was dehydrated by Na_2SO_4, evaporated under reduced pressure, dried under vacuum, and a white solid was obtained.

Yield: 177 mg (67.3 %)

^{1}H-NMR (300.40 MHz, $CDCl_3$): δ = 5.68 (s, 1H, 4-position), 4.98 (s, 1H, 21-position), 4.85 (s, 1H, 21-position), 4.47 (s, 1H, 11-position), 2.77 (t, 1H, 16-position), 2.48-2.17 (m, 5H, 1, 2, 6-position), 2.08-2.00 (m, 3H, 7, 8, 12-position), 2.05 (s, 3H, adamantane), 1.97 (s, 6H,

adamantane), 1.88 (m, 1H, 1-position), 1.86-1.76 (m, 2H, 14, 15-positon), 1.68 (t, 6H, adamantane), 1.56 (m, 3H, 12, 15, 16-position), 1.44 (s, 3H, 19-position), 1.06 (m, 2H, 7, 9-position), 0.98 (s, 3H, 18-position)
MALDI-TOF-MS: m/z=699.1 [$M+H^+$], 721.3 [$M+Na^+$]

3-2. Cortisol-3-oxime-O-acetic acid-21-adamantane carboxylate (3)

Compound 2 (131 mg, 0.25 mmol) was dissolved in MeOH / CH_2Cl_2 (1: 1) (5 mL), followed by the addition of pyrrolidine (124 µL, 1.5 mmol). After stirring for 20 min, the solution changed color to yellow. Carboxymethoxylamine hemihydrochloride (54.5 mg, 0.5 mmol) was then added, and the mixture was stirred at room temperature for 5 h. The solvent was evaporated under reduced pressure and pure water was added to the residue. The pH of the solvent was adjusted to 2.0 using a 1M HCl aqueous solution and extracted with EtOAc. The organic layer was washed with brine. The solvent was dehydrated using Na_2SO_4, evaporated under reduced pressure, and dried under vacuum, yielding a pale yellow solid.
Yield: 149 mg (99.7 %)
^{1}H-NMR (300.40 MHz, DMSO-d_6): δ = 5.71 (s, 1H, 4-position), 5.07-4.99 (m, 1H, 21-position), 4.85-4.78 (m, 1H, 21-position), 4.60-4.58 (m, 2H, -OC-CH2-O-), 4.44 (s, 1H, 11-position), 2.76 (m, 1H, 16-position), 2.42-2.17 (m, 5H, 1, 2, 6-position), 2.03 (m, 3H, 7, 8, 12-position), 1.97 (s, 3H, adamantane), 1.96 (s, 6H, adamantane), 1.73 (t, 6H, adamantane), 1.56-1.46 (m, 4H, 1, 12, 14, 15-position), 1.37-1.34 (m, 5H, 15, 16, 19-position), 1.09 (s, 1H, 9-position), 0.96 (m, 5H, 7, 9, 18-position)
MALDI-TOF-MS m/z=598.8 [$M+H^+$], 620.8 [$M+Na^+$]

3-3. Cortisol-3-oxime-{O-[N-(4-vinylphenyl)]-acetamide}-21-adamantane carboxylate (4) (TM1)

Compound 3 (149 mg, 0.25 mmol) was dissolved in MeOH (5 mL). 4-Aminostyrene (44.5 mg, 0.375 mmol) and DMT-MM (138 mg, 0.5 mmol) were added, followed by stirring the mixture overnight. The solvent was then evaporated under reduced pressure and dissolved in EtOAc. The solution was washed using a saturated citric acid aqueous solution, a saturated $NaHCO_3$ aqueous solution, and brine. The crude product was purified using preparative chromatography (AI-580S, Yamazen Corporation, Osaka, Japan) (silica gel, EtOAc : Hex = 1 : 2). The solvent was dehydrated by Na_2SO_4, evaporated under reduced pressure, dried under vacuum, yielding a white solid.
Yield: 61.0 mg (34.9 %)
^{1}H-NMR (300.40 MHz, $CDCl_3$): δ = 8.04 (m, 1H, NH), 7.52-7.49 (d, 2H, benzene ring),

7.39-7.36 (d, 2H, benzene ring), 6.72-6.63 (m, 1H, vinyl), 5.73-5.66 (m, 1H, 4-position), 5.22-5.18 (m, 1H, vinyl), 5.05-4.99 (m, 1H, vinyl), 4.84-4.78 (m, 1H, 21-position), 4.62-4.60 (d, 2H, 21-position, -OC-CH2-O-), 4.46 (s, 1H, 11-position), 2.77 (t, 1H, 16-position), 2.49-2.29 (m, 5H, 1, 2, 6-position), 2.07 (m, 3H, 7, 8, 12-position), 2.02 (s, 3H, adamantane), 1.97 (s, 6H, adamantane), 1.83-1.76 (m, 2H, 1, 14-positon), 1.73 (t, 6H, adamantane), 1.40-1.37 (m, 5H, 15, 16, 19-position), 1.07 (m, 2H, 7, 12-position), 0.98 (m, 4H, 9, 18-position)

MALDI-TOF-MS m/z=699.1 [$M+H^+$], 721.3 [$M+Na^+$]

4. Synthesis of 6-amino-*β*-cyclodextrin (6-NH_2-*β*-CD) and *N*-methacryloyl 6-amido-*β*-cyclodextrin (6-MAm-*β*-CD)

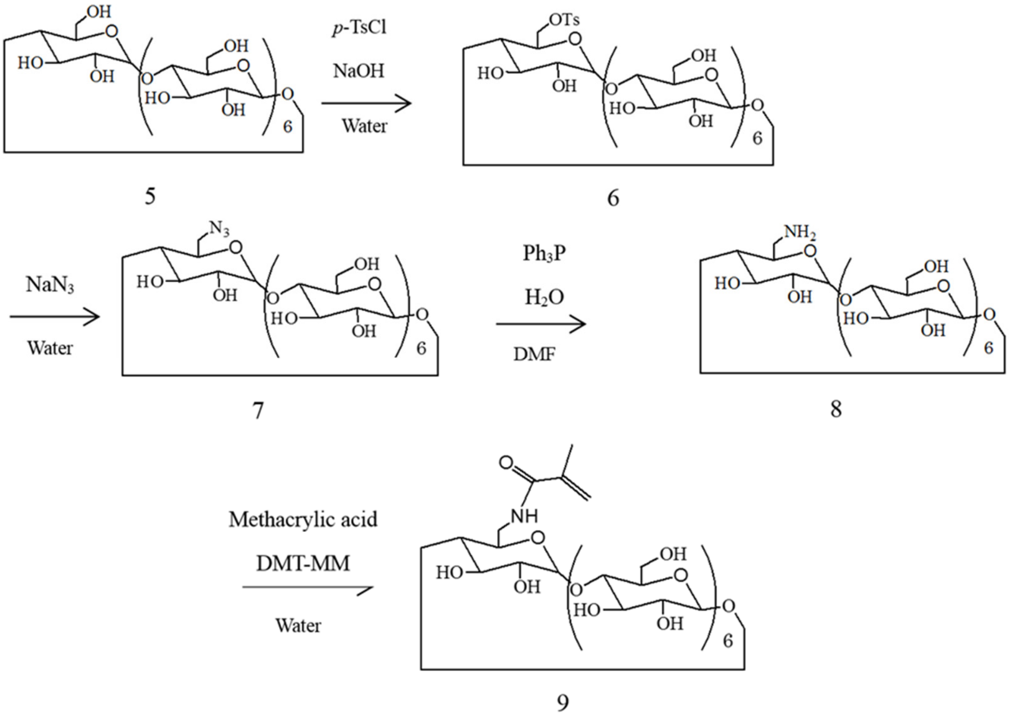

Scheme S2. Synthesis of 6-NH_2-*β*-CD and 6-MAm-*β*-CD

4-1. 6-TsO-*β*-CD (6)

Compound 5 (*β*-CD, 2.0 g, 1.75 mmol) was dissolved in a 0.4 M NaOH aqueous solution (40 mL) under cooling in an ice bath, and *p*-toluenesulfonyl chloride (2.0 g, 10.5 mmol) was added and stirred for 4 h. Unreacted *p*-toluenesulfonyl chloride was removed by filtration, and the filtrate was neutralized using a HCl aqueous solution. The filtrate was allowed to stand overnight, and the crystals grown were collected by vacuum filtration. Finally, the crystals were dried under vacuum, yielding a white solid.

Yield: 830 mg (36.8 %)
^{1}H-NMR (300.40 MHz, DMSO (D6)): δ = 7.76 (d, 2H, phenyl), 7.43 (d, 2H, phenyl), 5.89-5.57 (m, 14H, OH), 4.91-4.73 (m, 7H, CD), 4.52-4.31 (m, 7H, CD), 3.77 (m, 42H, CD), 2.43 (s, 3H, Methyl)
MALDI-TOF-MS: m/z=1311 [M+Na$^+$]

4-2. 6-N_3-*β*-CD (7)

Compound 6 (830 mg, 0.644 mmol) was suspended in water (10 mL) at 80 °C, NaN_3 (455 mg, 7 mmol) was added, and the mixture was stirring overnight at 80 °C. After the reaction was completed, the solution was slowly cooled to room temperature. Acetone (60 mL) was poured, and the resulting white precipitate was collected by filtration. The precipitate was dried under vacuum, yielding a white solid.
Yield: 548 mg (73.4 %)
^{1}H-NMR (300.40 MHz, DMSO (D6)): δ = 5.74-5.63 (m, 14H, OH), 4.82 (m, 7H, CD), 4.52-4.46 (m, 6H, CD), 3.62-3.57 (m, 42H, CD)
MALDI-TOF-MS: m/z=1182 [M+Na$^+$]

4-3. 6-NH_2-*β*-CD (8)

Compound 7 (548 mg, 0.472 mmol) was dissolved in DMF (5 mL), and Ph_3P (262 mg, 1.0 mmol) was added. After stirring the solution for 1 h at room temperature, pure water (1 mL) was added to the reaction mixture, followed by stirring overnight at 90 °C. After the reaction was completed, the solution was slowly cooled to room temperature. Acetone (60 mL) was poured, and the resulting white precipitate was collected by filtration. Finally, it was dried under vacuum, yielding a white solid.
Yield: 430 mg (80.4 %)
^{1}H-NMR (300.40 MHz, DMSO (D6)): δ = 5.76-5.65 (m, 14H, OH), 4.82 (m, 7H, CD), 4.46 (b, 6H, CD), 3.63-3.55 (m, 28H, CD)
MALDI-TOF-MS: m/z=1156 [M+Na$^+$]

4-4. 6-MAm-*β*-CD (9)

Compound 8 (430 mg, 0.38 mmol) and methacrylic acid (39 μL, 0.45 mmol) were dissolved in water (5 mL), and DMT-MM (221 mg, 0.8 mmol) was added. After stirring the solution overnight at room temperature, acetone (60 mL) was added and the precipitated white solid was corrected, and dried under vacuum.
Yield: 374 mg, 81.8 %

^{1}H-NMR (300Hz, D_2O) : δ =5.58 (s , 1H , methacryl) , 5.35 (s , 1H , methacryl) , 4.97-4.86 (m , H , CD) , 3.89-3.72 (m , H ,CD) , 3.52-3.35 (m , H , CD) , 1.79 (s , 3H , methyl)
MALDI-TOF-MS: m/z=1224 [M＋Na^+]

5. Synthesis of fluorescein-labeled bisphenol A (FITC-BPA)

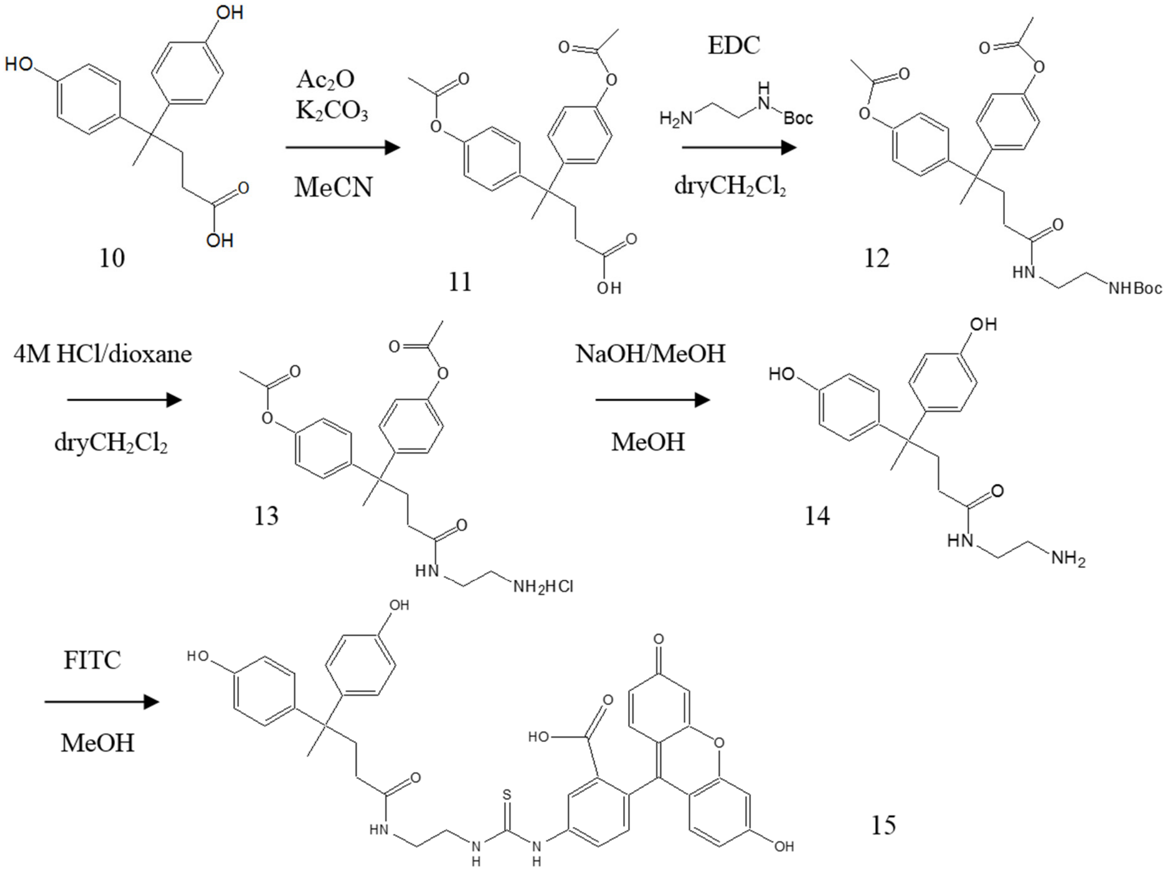

Scheme S3. Synthesis of fluorescein-labeled bisphenol A (FITC-BPA)

5-1. *N* -Boc-ethylenediamine

Ethylenediamine (1.75 mL, 25 mmol dissolved in CH_2Cl_2, 20 mL) and $(Boc)_2O$ (0.579 g, 2.65 mmol dissolved in CH_2Cl_2 (25 ml) were mixed using a dropping funnel under cooling with an ice bath. After stirring the solution for 6 h, the solvent was evaporated under reduced pressure and pure water was added for precipitating di-Boc-ethylenediamine. The precipitate was filtrated using a membrane filter (MF- Millipore cellulose mixed ester; pore size 0.22 μm), and a saturated Na_2CO_3 aqueous solution was added to the filtrate till basic (pH 10 - 11). After the extraction with CH_2Cl_2, the organic phase was dehydrated using $MgSO_4$, evaporated under reduced pressure, and dried under vacuum, yielding a white solid.

Yield: 387.6 mg (91.3 %)
^{1}H-NMR (300.40 MHz, $CDCl_3$) δ=4.87 (br,1H,-NH-), 3.18 (q,2H,-NH-CH_2-), 2.79 (t,2H,-CH_2-NH_2), 1.45 (s,9H,-O-C(CH_3)$_3$), 1.41 (s,2H,NH_2)

5-2. *O,O'*-diacetyl diphenolic acid (11)

Compound 10 (diphenolic acid, 1.43 g, 5 mmol) and K_2CO_3 (2.76 g, 20 mmol) were dissolved in MeCN (40 mL), and acetic anhydride (1.88 mL, 20 mmol) was added, followed by stirring overnight at 60 °C. The solvent was evaporated under reduced pressure, washed with brine, and extracted with EtOAc. Preparative chromatography (AI-580S, Yamazen Corporation, Osaka, Japan) was used for purification (silica gel, EtOAc : acetic acid = 90 : 10 : 1 → 70 : 30 : 1). The solvent was dehydrated by $MgSO_4$, evaporated under reduced pressure, and dried under vacuum, yielding a white solid.
Yield: 659 mg (35.6 %)
^{1}H-NMR (300.40 MHz, $CDCl_3$) δ = 7.18 (d, 4H, benzene ring), 7.00 (d, 4H, benzene ring), 2.42 (t, 2H, -CH_2-COOH), 2.29 (s, 6H, -$OCOCH_3$), 2.13 (t, 2H, -CH_2-CH_2-COOH), 1.62 (s, 3H, -CH_3)

5-3. *O,O'*-diacetyl diphenolic acid (2-Boc-aminoethyl)amide (12)

Compound 10 (390 mg, 1.05 mmol) and EDC (403 mg, 2.1 mmol) were dissolved in CH_2Cl_2 (10 mL), and *N* -Boc-ethylenediamine (252 mg, 1.58 mmol) was added, followed by stirring for 4 h under nitrogen atmosphere. The solvent was evaporated under reduced pressure and washed with brine. Preparative chromatography (AI-580S, Yamazen Corporation, Osaka, Japan) was used for purification (silica gel, EtOAc : CH_2Cl_2 = 3 : 7). The solvent was dehydrated by $MgSO_4$, evaporated under reduced pressure, and dried under vacuum, yielding a white solid.
Yield: 328 mg (61.0 %)
^{1}H-NMR (300.40 MHz, $CDCl_3$): δ = 7.19 (d, 4H, benzene ring), 6.99 (d, 4H, benzene ring),6.02 (s, 1H, -NH-), 4.91 (s, 1H, -NH-), 3.28 (m, 4H, -NH- (CH_2) $_2$-NH-),2.46 (t, 2H, -CH_2-COOH), 2.28 (s, 6H, -$OCOCH_3$),1.94 (t, 2H, -CH_2-CH_2-COOH), 1.60 (s, 3H, -CH_3), 1,41 (s, 9H, -NHCOO (CH_3) $_3$)

5-4. Diphenolic acid (2-aminoethyl)amide hydrochloride (13)

Compound 11 (225.8 mg, 0.44 mmol) was dissolved in CH_2Cl_2 (5 mL) under cooling with an ice bath, followed by the addition of 4M HCl / dioxane (450 μL, 1.76 mmol) slowly added and stirred overnight. The solution gradually turned yellow with time, and a solid was observed at the end of the reaction. The solvent was evaporated under reduced pressure, dried under vacuum.
Yield: 201 mg (98.4 %)

^{1}H-NMR (300.40 MHz, $CDCl_3$): δ = 7.14 (d, 4H, benzene ring), 6.94 (d, 4H, benzene ring), 2.23 (s, 6H, CH_3COO-), 2.08 (s, 3H, CH_3-), 1.53 (m, 4H, -CH_2-CH_2-), 1.25 (m, 4H, -CH_2-CH_2-)

5-5. Diphenolic acid (2-aminoethyl)amide (14)

Compound 13 (154.3 mg, 0.332 mmol) was dissolved in MeOH (5 mL) under cooling with an ice bath, followed by the addition of 0.2 M NaOH / MeOH solution (6.64 mL, 1.33 mmol) and stirring for 20 min. The reaction solution was evaporated under reduced pressure, washed with a saturated $NaHCO_3$ aqueous solution, and extracted with EtOAc. The solvent was dehydrated by $MgSO_4$, evaporated under reduced pressure, and dried under vacuum, yielding a white solid.

Yield: 61.8 mg (59.6 %)

^{1}H-NMR (300.40 MHz, DMSO (D6)): δ = 9.16 (s, 2H, -OH), 6.93 (d, 4H, benzene ring),6.64 (d, 4H, benzene ring), 2.99 (m, 4H, -NH-CH_2-CH_2-NH_2), 2.18 (m, 2H, -CH_2-CH_2-CO-),1.82 (m, 2H, -CH_2-CH_2-CO-), 1.45 (s, 3H, -CH_3)

5-6. Fluorescein-labeled bisphenol A (FITC-BPA) (15)

Compound 14 (32.0 mg, 0.1 mmol) and FITC (24.5 mg, 0.0630 mmol) were dissolved in MeOH (5 mL), followed by stirring overnight. Preparative chromatography (AI-580S, Yamazen Corporation, Osaka, Japan) was used for purification (silica gel, EtOAc : acetic acid = 100 : 1). The solvent was dehydrated by Na_2SO_4, evaporated under reduced pressure, and dried under vacuum, yielding a white solid.

Yield: 32.3 mg (71.4 %)

^{1}H-NMR (300.40 MHz, DMSO (D6)): δ = 10.1 (s, 1H, -COOH), 9.19 (s, 2H, -OH), 6.92 (d, 4H, benzene ring), 6.61 (m, 13H, H of the benzene ring and H of FITC), 3.25 (m, 4H, -NH-CH_2-CH_2-NH_2), 2.18 (m, 2H, -CH_2-CH_2-CO-), 1.77 (m, 2H, -CH_2-CH_2-CO-), 1.45 (s, 3H, -CH_3)

MALDI-TOF-MS: m/z=719.193 [M+H^+], 741.162 [M+Na^+]

6. Stability of the SAM and the *poly*(MPC) on the gold-coated glass substrates

A gold-coated glass substrate was washed with water and EtOH, and dried with N_2, then cleaned with a UV-O_3 cleaner (Bioforce Nanosciences) for 20 min. Then the substrate was immersed in an EtOH solution (5 mL) containing bis[2-(2-bromoisobutyryloxy) undecyl]disulfide (1.5 µL, 2.5 µmol) and 11-amino-1-undecanethiol hydrochloride (600 µg, 2.5 µmol) at 25 °C for 24 h.

Poly(MPC) was prepared on the SAM substrate. SI-ATRP was conducted with MPC (88.6 mg, 300 µmol) under the conditions described in the experimental section of the main text, without using MBAAm. After polymerization, the substrate was washed with a 1 M EDTA-4Na solution and water, and dried with N_2.

In order to examine the stability of the SAM and the *poly*(MPC) on the gold-coated glass substrates under the acidic conditions employed for removal of the cortisol-21-adamantane carboxylate moiety by hydrolyzing the oxime bond, the substrates were immersed into a 100 mM aqueous HCl solution for 12 h at 40 °C, and the changes in the thicknesses of the SAM and the *poly*(MPC) before and after the HCl treatment were estimated by X-ray reflectometry (XRR).

Table S1. Thicknesses of the SAM and the *poly*(MPC) on the gold-coated glass substrates before and after HCl treatment

Entry	Thickness [nm]		
	Before	After	Delta
mixed SAM#1	2.21	2.31	0.10
mixed SAM#2	2.13	2.18	0.05

Entry	Thickness [nm]		
	Before	After	Delta
poly(MPC)#1	5.34	5.14	-0.20
poly(MPC)#2	5.02	4.94	-0.08

Almost no change is observed after the HCl treatment, confirming that the samples are stable under the conditions used for removal of the cortisol-21-adamantane carboxylate moiety.

7. Preparation of a NIP thin layer for examination of the imprinting effect

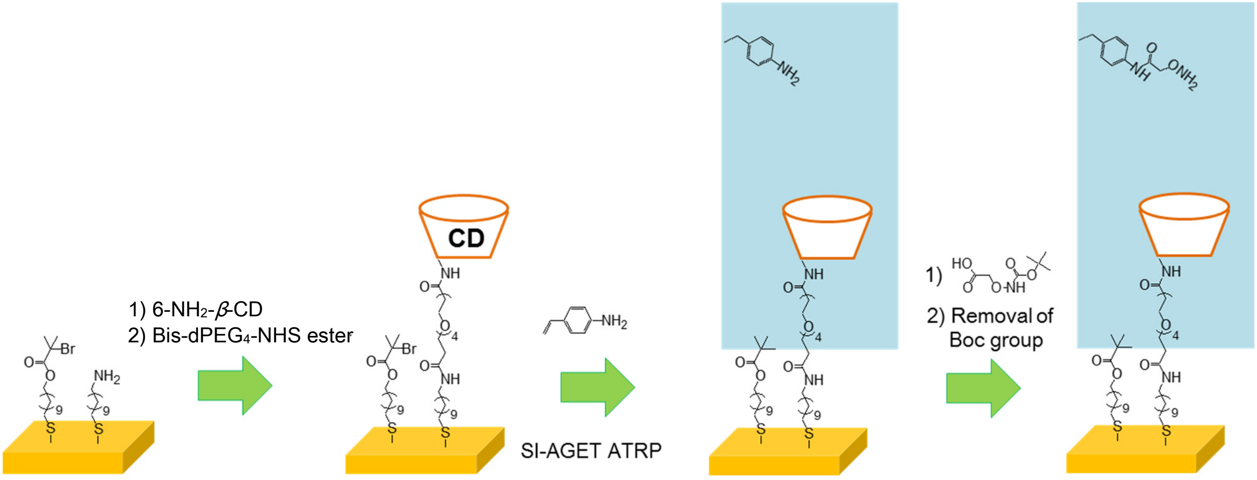

Scheme S4. Preparation of NIP with randomly located aminooxy groups that have no template molecule-induced binding cavity on the *β*-CD-immobilized gold-coated glass substrate.

NIP with no template molecule-induced binding cavity was prepared as a reference polymer for examination of the imprinting effect. SI-AGET ATRP was conducted on the β-CD-immobilized gold-coated glass substrate under the conditions described in the experimental section of the main text, using 4-amino styrene (0.715 mg, 6 μmol) instead of TM1, without the use of MBAAm. After polymerization, the substrate was washed with pure water and EtOH. N-Boc-2-(aminooxy)acetic acid (10.5 mg, 0.06 mmol) was then coupled with the 4-aminostyrene residues of the resulting polymer in DMF (6 mL) containing EDC (10.5 mg, 0.06 mmol) and DMAP (7.33 mg, 0.06 mmol) for 18 h at 25 °C. The substrate was then immersed in a 4 N HCl dioxane solution for 15 min at 25 °C to remove the Boc group, resulting in a NIP thin layer with randomly located aminooxy groups on the *β*-CD-immobilized substrate. The substrate was washed with pure water and EtOH, and dried with N_2.

8. Preparation of an RP-*β*-CD thin layer for examination of the effect of two point cortisol binding

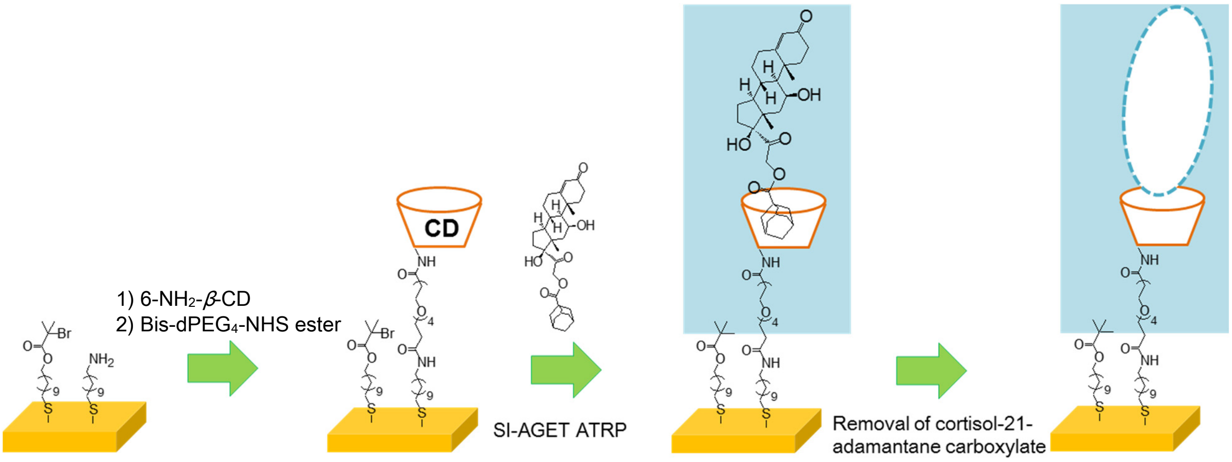

Scheme S5. Preparation of RP-*β*-CD with only β-CD inside its cavity.

RP-*β*-CD was prepared as a reference polymer to aid in examining the effect of two point-binding. In this case, RP-*β*-CD was prepared using cortisol-21-adamantane carboxylate (Compound 2 in Scheme S1), instead of TM1. Compound 2 (1.31 mg, 2.5 μmol) was dissolved in DMSO (200 μL), and pure water was added (2.5 mL). The *β*-CD-immobilized substrate was immersed in the DMSO solution for 24 h at 25 °C. The substrate was placed in a Schlenk flask and SI-AGET ATRP was conducted under the conditions described in the experimental section of the main text, without using MBAAm. After polymerization, the substrate was washed with pure water and immersed in a 1 M EDTA-4Na aqueous solution to remove Cu (II) ions at 25 °C for 12 h. The substrate was washed with pure water and EtOH, and dried with N_2.

9. Preparation of an RP-oxime thin layer to examine the effect of two point cortisol binding

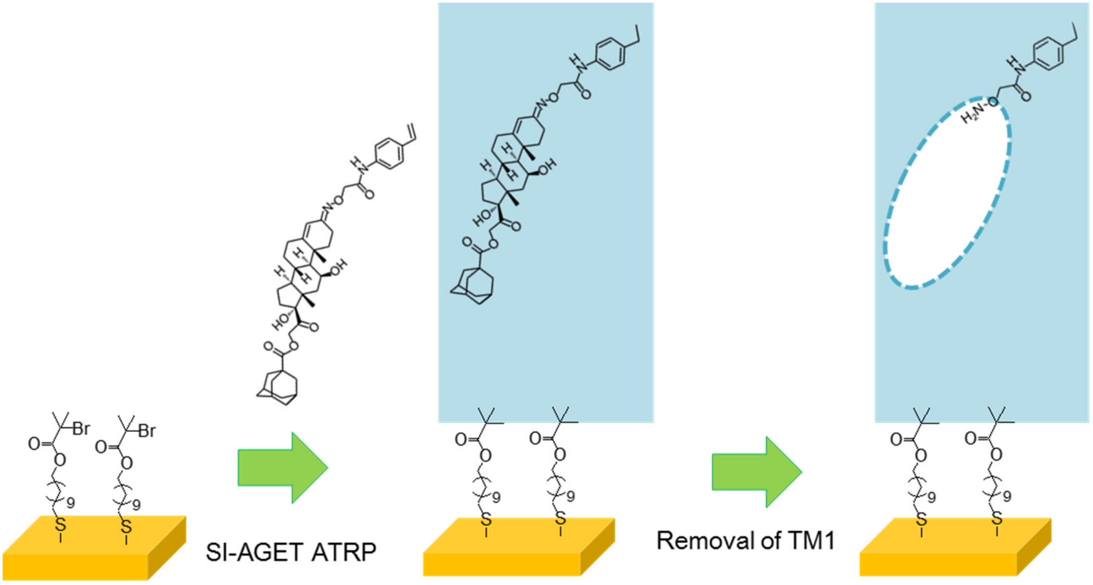

Scheme S6. Preparation of RP-oxime with only aminooxy groups inside its cavity.

RP-oxime was prepared as a reference polymer to aid in examining the effect of two point-binding. In this case, RP-oxime was prepared without immobilizing 6-NH_2-β-CD on the substrate. The substrate was placed in a Schlenk flask and SI-AGET ATRP was conducted under the conditions described in the experimental section of the main text, without using MBAAm. After polymerization, the substrate was washed with pure water and immersed in a 1 M EDTA-4Na aqueous solution to remove Cu (II) ions at 25 °C for 12 h. The substrate was then immersed into a 100 mM HCl aqueous solution at 40 °C for 12 h to remove the cortisol-21 adamantane carboxylate moiety by hydrolysis of the oxime bond. The substrate was washed with pure water and EtOH, and dried with N_2.

10. Preparation of an R-MIP thin layer to examine the effect of orientational immobilization of TM1

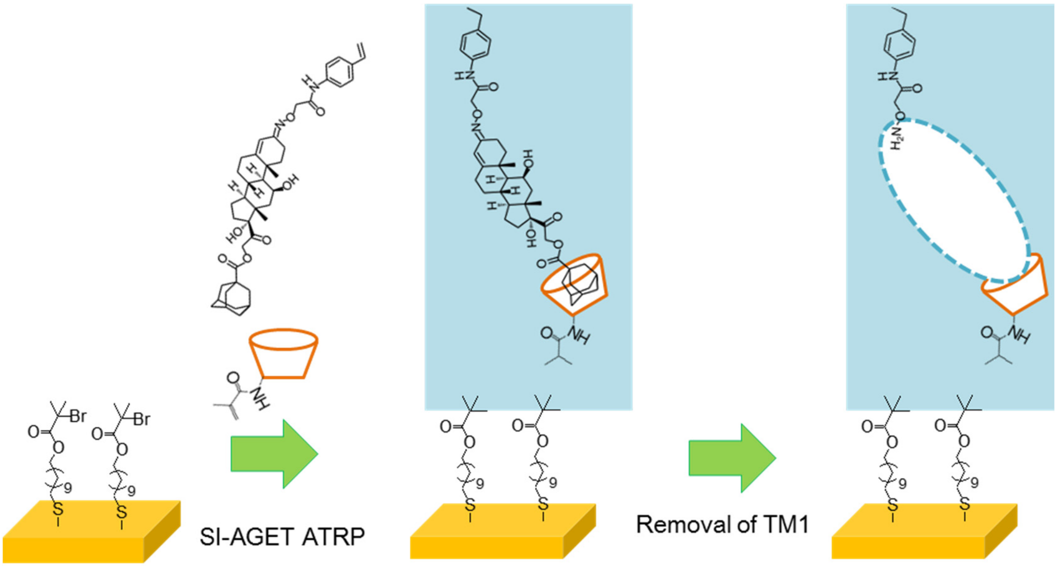

Scheme S7. Preparation of R-MIP with randomly located binding cavities that contain both *β*-CD and aminooxy groups.

R-MIP was prepared as a reference polymer to aid in examining the effect of the oriented immobilization of TM1 during SI-AGET ATRP. R-MIP was prepared on the substrate without immobilizing 6-NH_2-*β*-CD, and 6-MAm-*β*-CD was used instead of immobilized *β*-CD. SI-AGET ATRP was conducted using 6-MAm-*β*-CD (0.72 mg, 0.6 µmol), MPC (88.6 mg, 300 µmol), and TM1 (0.42 mg, 0.6 µmol) under the conditions described in the experimental section of the main text, without using MBAAm. After polymerization, the substrate was washed with pure water and immersed in a 1 M EDTA-4Na aqueous solution to remove Cu (II) ions at 25 °C for 12 h. The substrate was then immersed in a 100 mM HCl aqueous solution at 40 °C for 12 h to remove the cortisol-21 adamantane carboxylate moiety by hydrolysis of the oxime bond. The substrate was washed with pure water and EtOH, and dried with N_2.

11. Preparation of a *poly*(MPC) thin layer

Poly(MPC) was prepared on the substrate without immobilizing 6-NH_2-*β*-CD to aid in examining the effect of the polymer matrix on cortisol binding. SI-AGET ATRP was conducted with MPC (88.6 mg, 300 µmol) under the conditions described in the experimental section of the main text, without using MBAAm. After polymerization, the substrate was washed with water and EtOH, and dried with N_2.

12. Interaction between the adamantyl group of TM1 and β-CD

TM1 (10 mM) and β-CD (0, 10, and 50 mM) were dissolved in DMSO-d_6 (500 μL). ^{1}H-NMR spectra of these samples were obtained by 400-MHz FT-NMR apparatus (VarianUNITYNOVA400, Agilent Technologies Japan, Ltd., Tokyo, Japan).

The interaction between β-CD and the adamantane group of TM1 was confirmed by the peak shift to a high magnetic field side at around 2 ppm derived from the adamantyl group of TM1, while no interaction was observed at around 7.6 ppm derived from the styryl group of TM1.

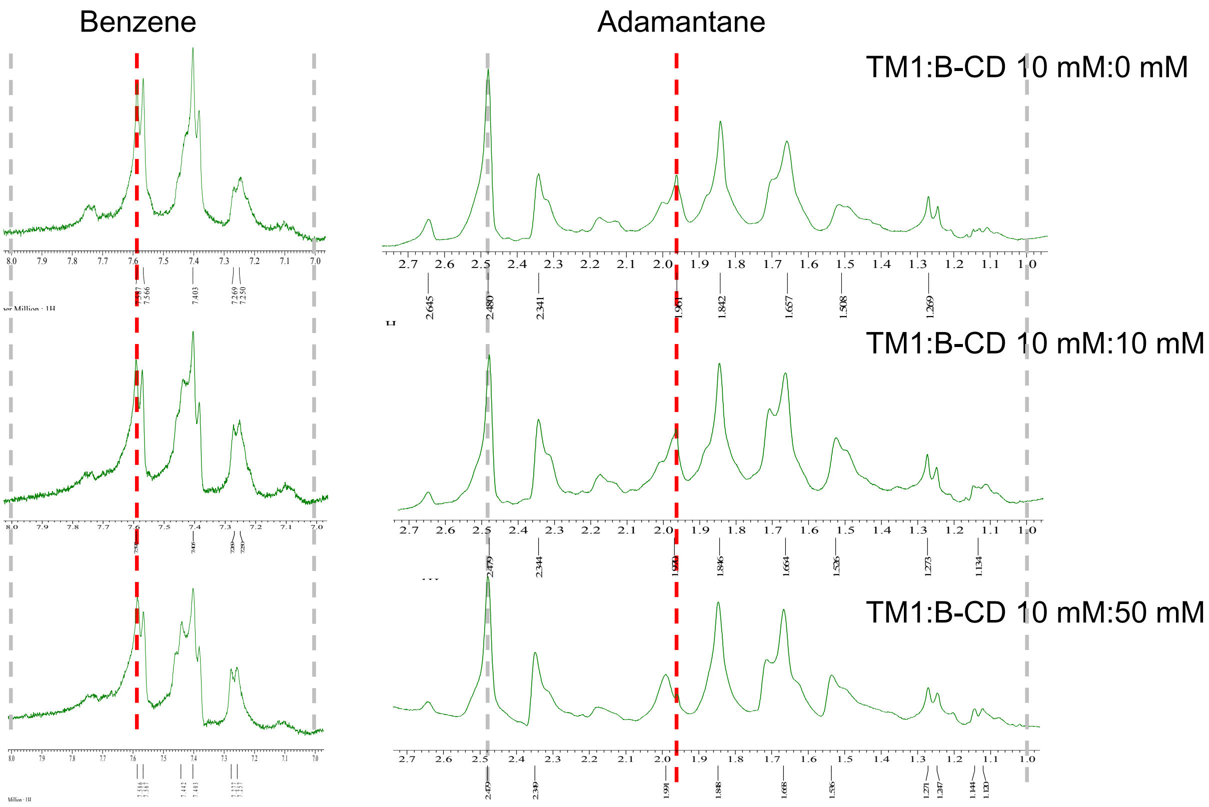

Figure S1. ^{1}H-NMR spectra for mixtures of TM1 (10 mM) and β-CD (0, 10, and 50 mM).

13. XPS measurements

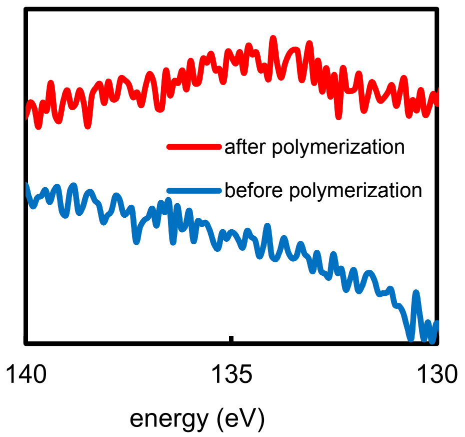

Figure S2. XPS spectra of P 2*p* orbital for before (blue) and after (red) SI-AGET ATRP for preparing the MIP thin layer

14. XRR measurements and the estimation of polymer thickness

(a) Gold-coated glass substrate

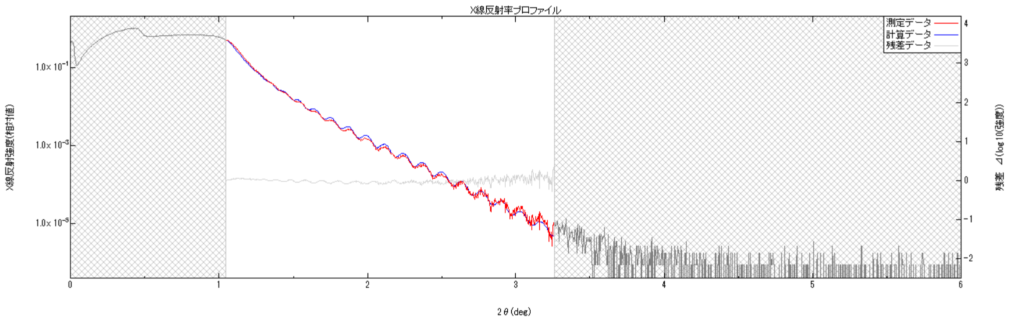

(b) TM1-immobilized gold-coated glass substrate

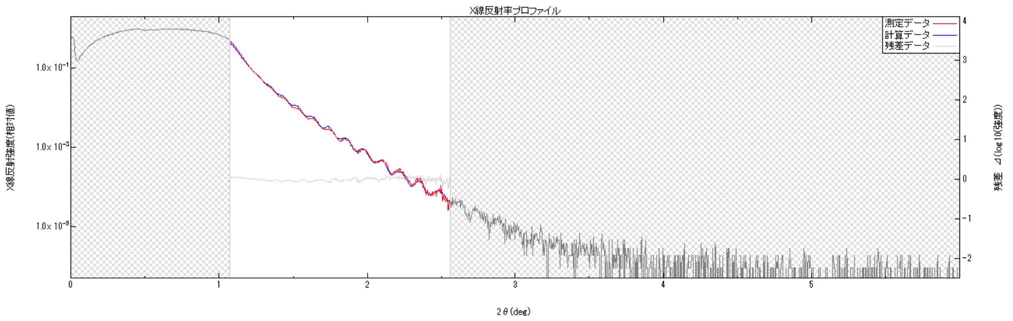

(c) The MIP-coated substrate prepared by SI-AGET ATRP for 30 min at 25 °C

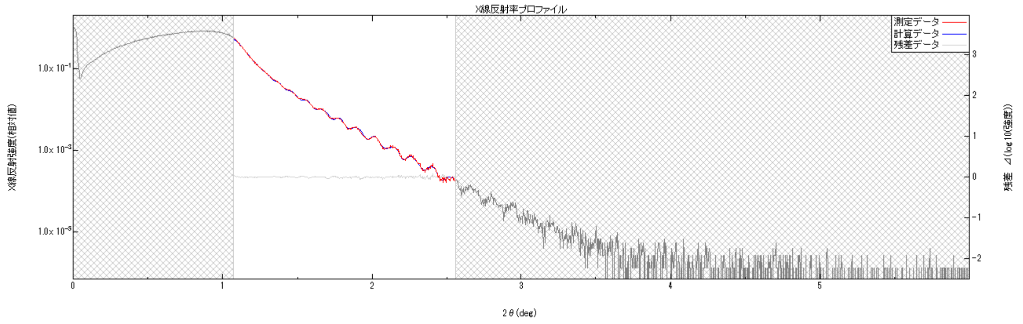

(d) The MIP-coated substrate prepared by SI-AGET ATRP for 1 h at 25 °C

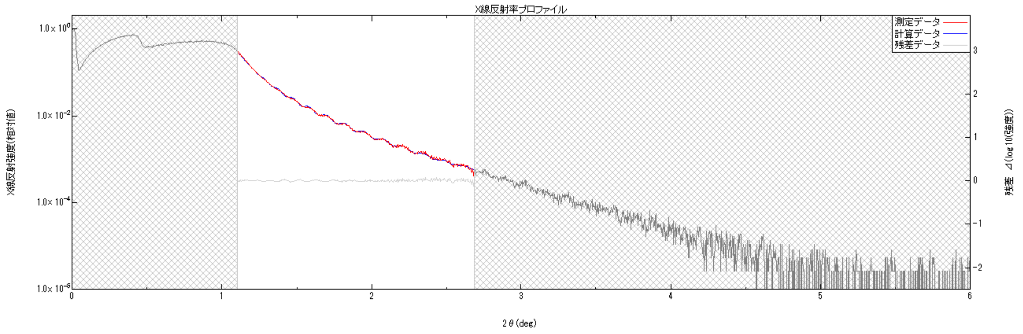

(e) The MIP-coated substrate prepared by SI-AGET ATRP for 3 h at 25 °C

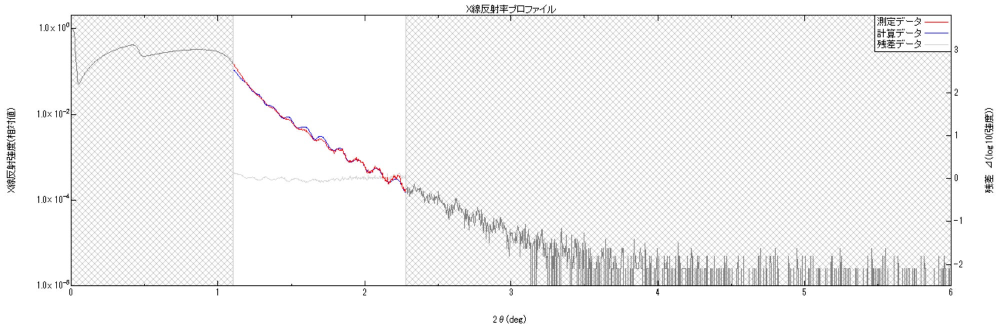

Figure S3. Fitting data of XRR measurements for the gold-coated glass substrate (a); TM1-immobilized gold-coated glass substrate (b); the MIP-coated gold-coated glass substrate prepared by SI-AGET ATRP at 25 °C for 30 min (c), 1 h (d), and 3 h (e).

Table S2. Calculation results of each substrate

	Layer Name	Thickness (nm)	Density (g/cm^3)	Roughness (nm)
**Au bare**	Au	60.62	18.7	0.9909
	Cr	40	7.2	1.139
	SiO_2	0	2.2	2
**TM1-immobilized substrate**	TM1-SAM	2.26	1.58	0
	Au	61.59	18.95	1.008
	Cr	39.9	7.2	1.32
	SiO_2	0	2.2	1.53
**MIP (25 °C, 30 min)**	PMPC	2.91	1.06	1.37
	SAM	2.04	1.66	0.225
	Au	60.3	18.9	0.582
	Cr	40.7	7.3	1.38
	SiO_2	0	2.2	1.48
**MIP (25 °C, 1 h)**	PMPC	6.48	1.06	1.87
	SAM	2.04	1.66	0.208
	Au	57.1	18.5	0.516
	Cr	41.2	7.37	1.36
	SiO_2	0	2.2	2.84
**MIP (25 °C, 3 h)**	PMPC	14.1	1.06	1.63
	SAM	2.04	1.66	0.729
	Au	60.1	15.7	0.984
	Cr	41.6	7.3	1.26
	SiO_2	0	2.2	1.66

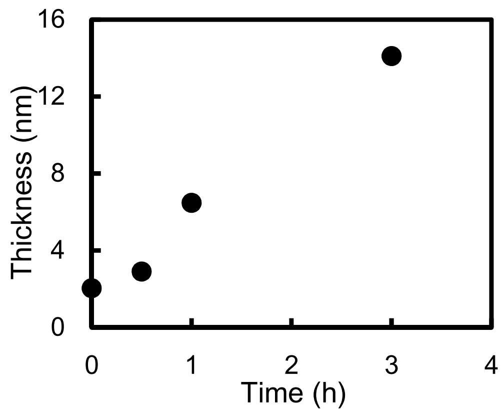

Figure S4. Time course of the thickness of MIP during SI-AGET ATRP of MPC at 25 °C.

15. Estimation of possible *β*-CD complex sizes with FITC-BPA and TM1

Possible conformations of *β*-CD complexes of FITC-BPA and TM1 were estimated using a molecular mechanics-based docking simulation under aqueous conditions (dielectric constant = 80) in MOE (Chemical Computing Group Inc., Montreal, Canada). It appears the sizes are similar, as shown in Figure S4.

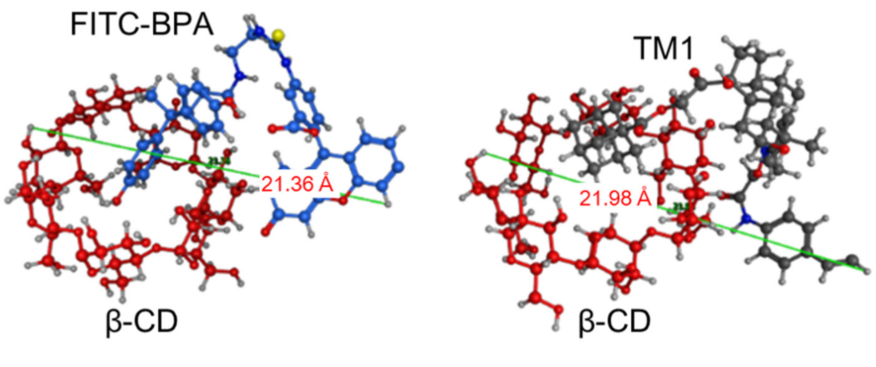

Figure S5. Possible conformations of *β*-CD (red) complexes of FITC-BPA (blue) and TM1 (Gray).

16. Time course of FITC-BPA adsorption to MIP on the substrate

FITC-BPA (100 nM) dissolved in a 10 mM phosphate buffer (pH 7.4) was transferred into a vial (5 mL), into which an MIP-coated substrate was inserted. The time evolution of the fluorescence intensity in the supernatant was measured at 0, 10, 20, 40, and 50 min at 25 °C (excitation wavelength: 495 nm, emission wavelength: 515 nm).

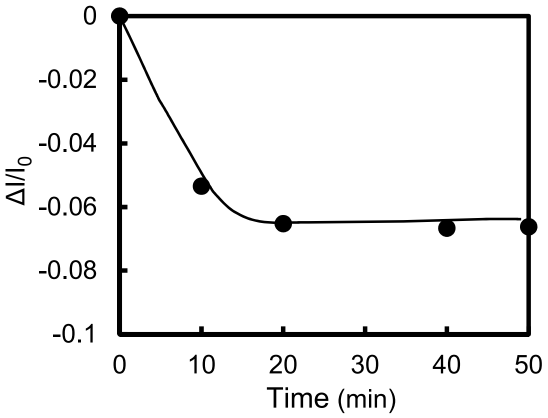

Figure S6. Time evolution of adsorption of FITC-BPA (100 nM) by the MIP thin layer.

17. Adsorption of FITC-BPA by MIP

Phosphate buffer solutions (10 mM, pH 7.4) containing various concentrations of FITC-BPA (final concentrations: 0 - 400 nM) were transferred into vials (5 mL), into which MIP-substrates were immersed. The vials were then incubated for 1 h at 25 °C. FITC-BPA (100 nM) dissolved in a phosphate buffer was then added, and after 20 min at 25 °C, fluorescence was measured at the same temperature (excitation wavelength: 495 nm, emission wavelength: 515 nm).

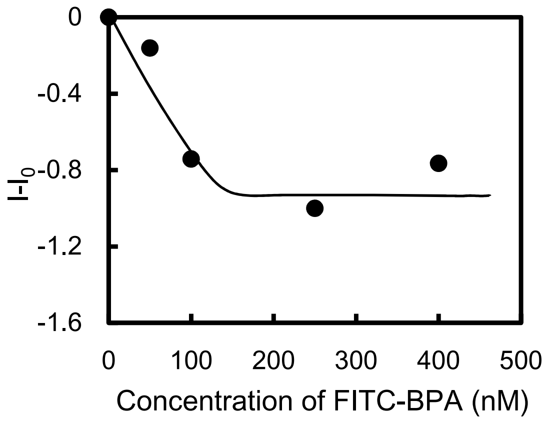

Figure S7. Adsorption of FITC-BPA to the MIP thin layer.

18. Interaction between FITC-BPA and cortisol

FITC-BPA (100 nM) dissolved in a 10 mM phosphate buffer (pH 7.4) was transferred into a vial (5 mL). This was followed by addition of cortisol dissolved in a phosphate buffer (final concentrations: 0 nM - 0.4 nM). After 20 min at 25 °C, fluorescence was measured at the same temperature (excitation wavelength: 495 nm, emission wavelength: 515 nm).

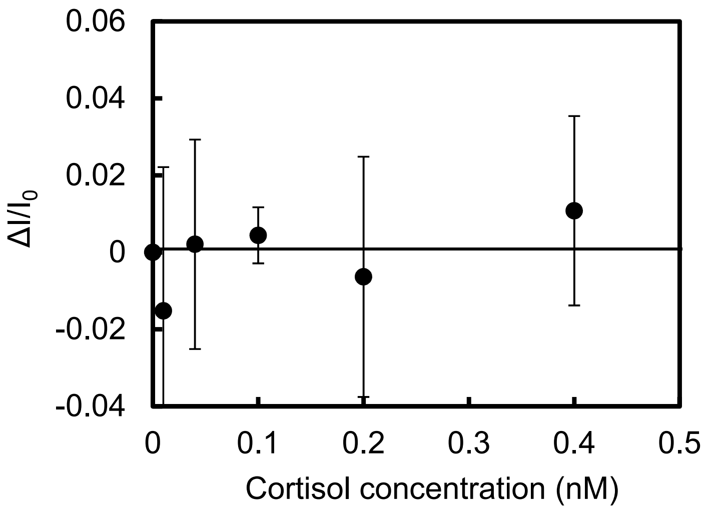

Figure S8. Changes in the relative fluorescence intensity by interaction with FITC-BPA and cortisol.

19. Time course of the replacement of FITC-BPA with cortisol

FITC-BPA (100 nM) dissolved in a 10 mM phosphate buffer (pH 7.4) was transferred into a vial (5 mL), into which MIP-substrates were immersed. The vials were then incubated for 1 h at 25 °C. Cortisol (0.1 nM) dissolved in the phosphate buffer was then added, and the time evolution of the fluorescence intensity of the supernatant was measured at 0, 10, 20, 30, and 40 min at 25 °C (excitation wavelength: 495 nm, emission wavelength: 515 nm).

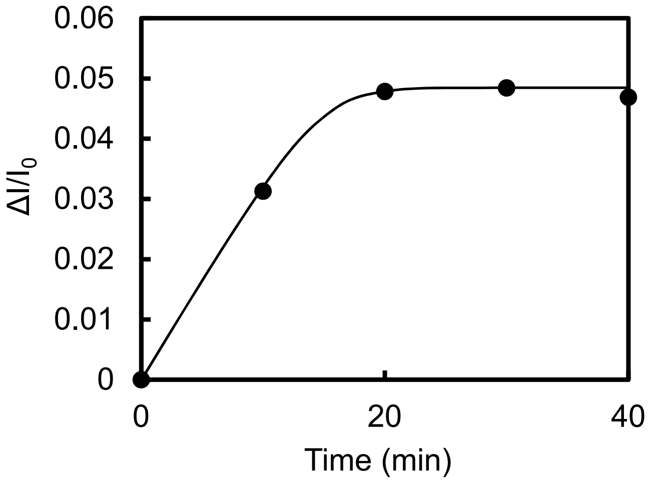

Figure S9. Time course of the replacement of FITC-BPA by cortisol on the MIP thin layer.

20. Fluorescence-based competitive binding assay for cortisol using MIPs, NIP, and a *poly*(MPC) thin layer

FITC-BPA (100 nM) dissolved in a 10 mM phosphate buffer (pH 7.4) was transferred into vials (5 mL), into which polymer-coated substrates were immersed. The vials were then incubated for 1 h at 25 °C. Cortisol dissolved in a 10 mM phosphate buffer (pH 7.4) (final concentrations: 0 nM – 2.0 nM) was added. Fluorescence was measured after 20 min at 25 °C (excitation wavelength: 495 nm, emission wavelength: 515 nm).

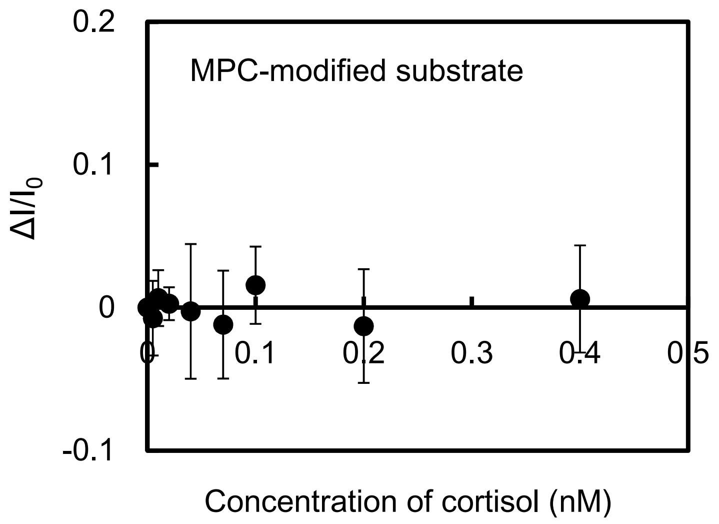

Figure S10. Changes in the relative fluorescence intensity by addition of cortisol to the MPC-modified substrate, as detected by the fluorescence-based competitive binding assay with FITC-BPA as the competitor (n=3).

The binding constant for cortisol was estimated by curve fitting using DeltaGraph 5.4.5v. The fitting equation is shown below. It is generally used with the binding constant for 1:1 complex formation, where Y is $\Delta I/I_0$, K is a binding constant, H is found by fitting raw data to a theoretical curve, G is a cortisol concentration, and D is the maximum amount of cortisol bound.

$$\mathrm{Y} = [(1 + KG + KH) - \sqrt{(1 + KG + KH) - 4K^2HG}] \times \frac{D}{2KG}$$

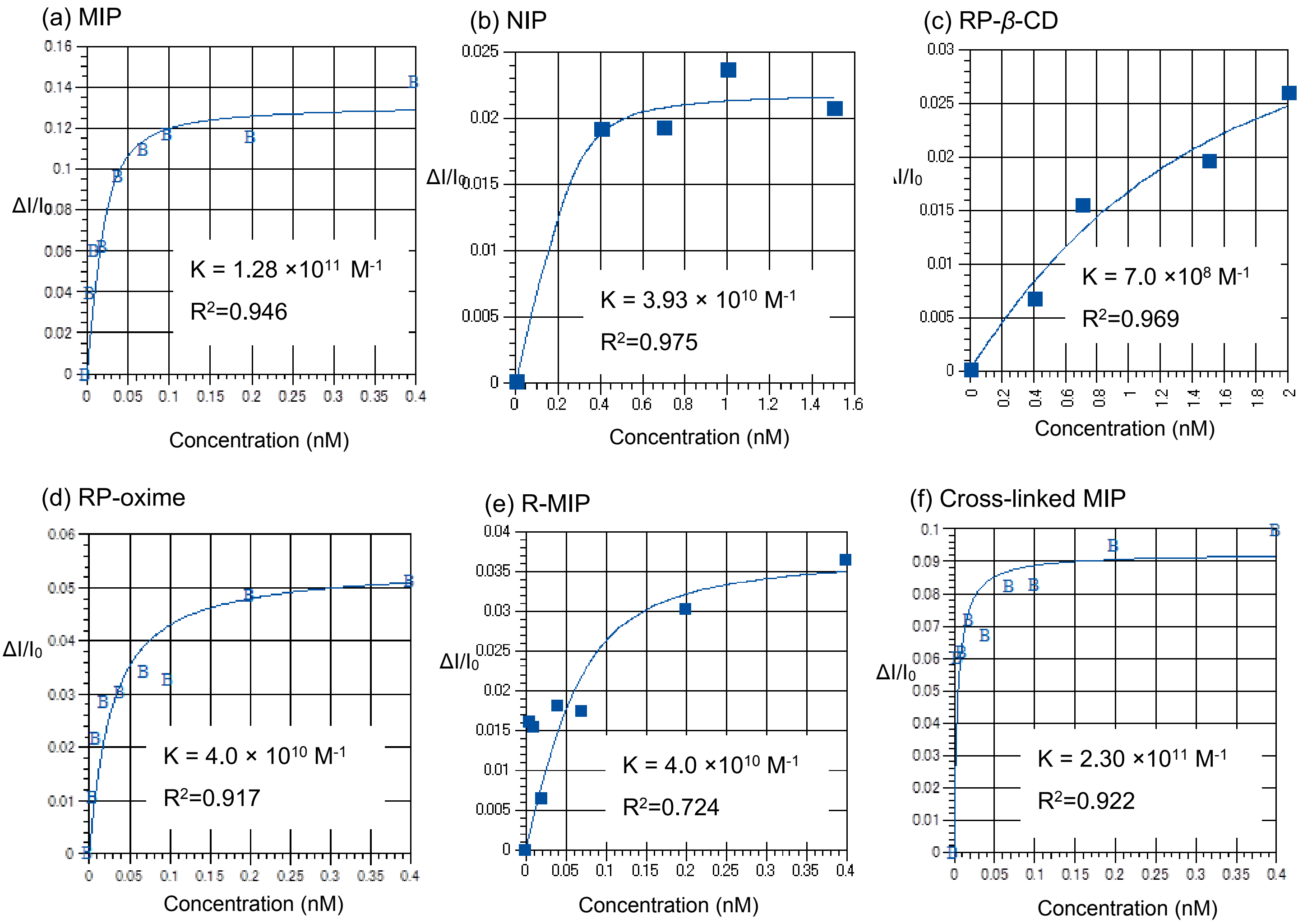

Figure S11. Curve fitting data from the fluorescent competitive binding assay of cortisol using MIP (a), RP-*β*-CD (b), RP-oxime (c), NIP (d), R-MIP (e), and the cross-linked MIP (f).

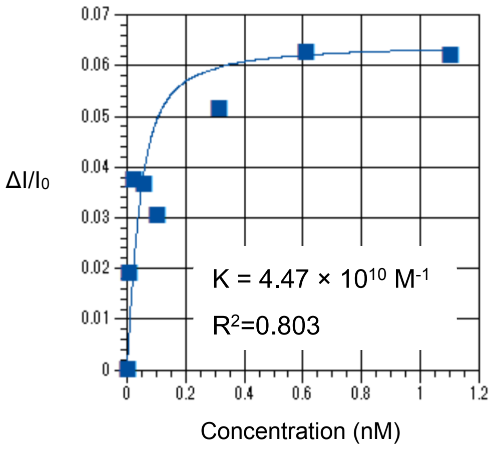

Figure S12. Curve fitting data from the fluorescent competitive binding assay of cortisol with the cross-linked MIP in 10% saliva.
